# Supplementary material for: “I want to help my body”: Acceptability of malaria chemoprevention among children with sickle cell anaemia and their caregivers in Malawi and Uganda
Source: PLOS Glob Public Health. 2025 Jul 9;5(7):e0004056. doi: 10.1371/journal.pgph.0004056 (PMC12240377; doi:10.1371/journal.pgph.0004056)
Supplement: Attachment — Submitted filename: Review of Manuscript.docx [file pgph.0004056.s002.docx]

**Review of Manuscript:”I want to help my body": Acceptability of malaria chemoprevention among children with sickle cell anaemia and their caregivers in Malawi and Uganda”**

This acceptability study should have been presented after the efficacy study. It would be preferable to discuss the efficacy and safety of chemoprevention first. Giving PD on a weekly basis seems too burdensome when we know that PD has a long shelf-life, especially piperaquine (more than 20 days in the blood). Secondly, the study makes no mention of a first dose taken under TDO, which is generally de rigueur when taking these long-lasting drugs.

Also, this study lacks a contextual analysis. There is no reminder of the epidemiology of malaria in Malawi and Uganda to understand the periodicity of chemoprevention administration. For whenever chemoprevention is used, it responds to the duration of the malaria transmission season, but not on an annual basis, as is the case here. The article does not mention whether CMS is implemented in these two countries, as the age range of the children in the study includes a large proportion of the CMS target group (6 months to 5 years).
