# Supplementary material for: “I want to help my body”: Acceptability of malaria chemoprevention among children with sickle cell anaemia and their caregivers in Malawi and Uganda
Source: PLOS Glob Public Health. 2025 Jul 9;5(7):e0004056. doi: 10.1371/journal.pgph.0004056 (PMC12240377; doi:10.1371/journal.pgph.0004056)
Supplement: Attachment — Submitted filename: Revision.docx [file pgph.0004056.s004.docx]

156-165: Use data from the latest WHO 2024 report.

320: had completed 5-7 grades of schooling (51.4%). This information is not found in Table 2.

336. Please review the way of presenting the tables should have only three horizontal lines in its presentation. Two lines to frame the labels and one line at the end of the table.

In Table 3, do you talk about the number of children with SCD or SCA?

382-385: Please provide a verbatim statement from your survey to argue the fact that "a few participants argued that weekly drugs may provide better protection against..."

386-396: Please review the title and content. Here, the verbatim statement you provided mainly indicates the difficulty of going to the hospital to get the medication.

569-571: “To our knowledge, this is the first study assessing acceptability towards weekly single-day treatment courses of DP. Weekly dosing of DP is predicted to be more effective at preventing malaria episodes than monthly dosing…” **In your results section you should provide more evidence to support what you are saying here.**

575-577: “In the final phase of data collection, we recruited a sub-sample of participants who had received weekly DP for an average period of 15 months as part of the parent trial. The retrospective phase was performed after trial finalisation and unbinding.” **We did not feel in the presentation of your results a presentation according to the different phases of your data collection.**
